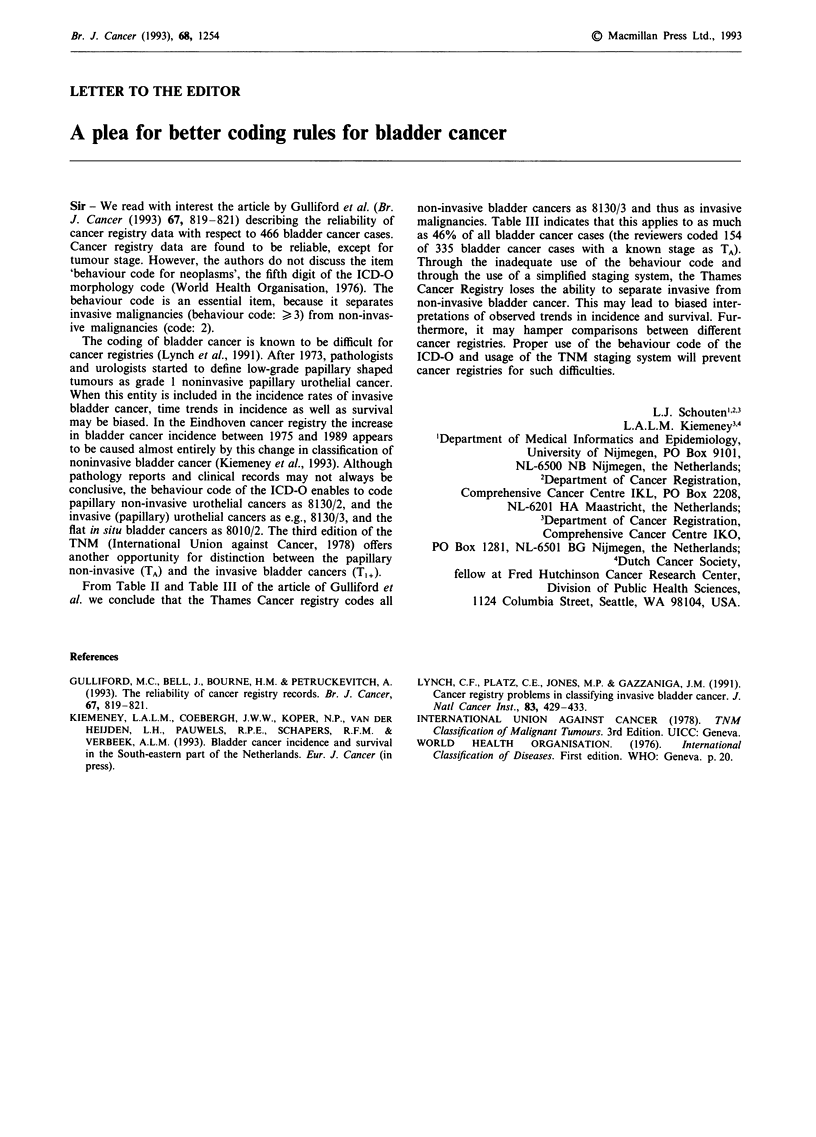# A plea for better coding rules for bladder cancer.

**DOI:** 10.1038/bjc.1993.514

**Published:** 1993-12

**Authors:** L. J. Schouten, L. A. Kiemeney


 © Macmillan Press Ltd., 1993

LETTER TO THE EDITOR

### A plea for better coding rules for bladder cancer

**Sir** – We read with interest the article by Gulliford *et al.* (*Br. J. Cancer* (1993) **67**, 819–821) describing the reliability of cancer registry data with respect to 466 bladder cancer cases. Cancer registry data are found to be reliable, except for tumour stage. However, the authors do not discuss the item 'behaviour code for neoplasms', the fifth digit of the ICD-O morphology code (World Health Organisation, 1976). The behaviour code is an essential item, because it separates invasive malignancies (behaviour code: $\geqslant 3$) from non-invasive malignancies (code: 2).

The coding of bladder cancer is known to be difficult for cancer registries (Lynch *et al.*, 1991). After 1973, pathologists and urologists started to define low-grade papillary shaped tumours as grade 1 noninvasive papillary urothelial cancer. When this entity is included in the incidence rates of invasive bladder cancer, time trends in incidence as well as survival may be biased. In the Eindhoven cancer registry the increase in bladder cancer incidence between 1975 and 1989 appears to be caused almost entirely by this change in classification of noninvasive bladder cancer (Kiemeney *et al.*, 1993). Although pathology reports and clinical records may not always be conclusive, the behaviour code of the ICD-O enables to code papillary non-invasive urothelial cancers as 8130/2, and the invasive (papillary) urothelial cancers as e.g., 8130/3, and the flat *in situ* bladder cancers as 8010/2. The third edition of the TNM (International Union against Cancer, 1978) offers another opportunity for distinction between the papillary non-invasive ($T_A$) and the invasive bladder cancers ($T_{1+}$).

From Table II and Table III of the article of Gulliford *et al.* we conclude that the Thames Cancer registry codes all non-invasive bladder cancers as 8130/3 and thus as invasive malignancies. Table III indicates that this applies to as much as 46% of all bladder cancer cases (the reviewers coded 154 of 335 bladder cancer cases with a known stage as $T_A$). Through the inadequate use of the behaviour code and through the use of a simplified staging system, the Thames Cancer Registry loses the ability to separate invasive from non-invasive bladder cancer. This may lead to biased interpretations of observed trends in incidence and survival. Furthermore, it may hamper comparisons between different cancer registries. Proper use of the behaviour code of the ICD-O and usage of the TNM staging system will prevent cancer registries for such difficulties.

L.J. Schouten[1,2,3]
L.A.L.M. Kiemeney[3,4]
[1]Department of Medical Informatics and Epidemiology, University of Nijmegen, PO Box 9101, NL-6500 NB Nijmegen, the Netherlands;
[2]Department of Cancer Registration, Comprehensive Cancer Centre IKL, PO Box 2208, NL-6201 HA Maastricht, the Netherlands;
[3]Department of Cancer Registration, Comprehensive Cancer Centre IKO, PO Box 1281, NL-6501 BG Nijmegen, the Netherlands;
[4]Dutch Cancer Society, fellow at Fred Hutchinson Cancer Research Center, Division of Public Health Sciences, 1124 Columbia Street, Seattle, WA 98104, USA.

### References

GULLIFORD, M.C., BELL, J., BOURNE, H.M. & PETRUCKEVITCH, A. (1993). The reliability of cancer registry records. *Br. J. Cancer*, **67**, 819–821.

KIEMENEY, L.A.L.M., COEBERGH, J.W.W., KOPER, N.P., VAN DER HEIJDEN, L.H., PAUWELS, R.P.E., SCHAPERS, R.F.M. & VERBEEK, A.L.M. (1993). Bladder cancer incidence and survival in the South-eastern part of the Netherlands. *Eur. J. Cancer* (in press).

LYNCH, C.F., PLATZ, C.E., JONES, M.P. & GAZZANIGA, J.M. (1991). Cancer registry problems in classifying invasive bladder cancer. *J. Natl Cancer Inst.*, **83**, 429–433.

INTERNATIONAL UNION AGAINST CANCER (1978). *TNM Classification of Malignant Tumours*. 3rd Edition. UICC: Geneva.

WORLD HEALTH ORGANISATION. (1976). *International Classification of Diseases*. First edition. WHO: Geneva. p. 20.